# What Is Unfair about Unequal Brute Luck? An Intergenerational Puzzle

**DOI:** 10.1007/s11406-018-00053-5

**Published:** 2019-01-21

**Authors:** S. J. Beard

**Affiliations:** Centre for the Study of Existential Risk, University of Cambridge, Cambridge, UK

**Keywords:** Luck egalitarianism, Unfairness, Intergenerational justice

## Abstract

According to Luck egalitarians, fairness requires us to bring it about that nobody is worse off than others where this results from brute bad luck, but not where they choose or deserve to be so. In this paper, I consider one type of brute bad luck that appears paradigmatic of what a Luck Egalitarian ought to be most concerned about, namely that suffered by people who are born to badly off parents and are less well off as a result. However, when we consider what is supposedly unfair about this kind of unequal brute luck, luck egalitarians face a dilemma. According to the standard account of luck egalitarianism, differential brute luck is unfair because of its effects on the distribution of goods. Yet, where some parents are worse off because they have chosen to be imprudent, it may be impossible to neutralize these effects without creating a distribution that seems at least as unfair. This, I argue, is problematic for luck egalitarianism. I, therefore, explore two alternative views that can avoid this problem. On the first of these, proposed by Shlomi Segall, the distributional effects of unequal brute luck are unfair only when they make a situation more unequal, but not when they make it more equal. On the second, it is the unequal brute luck itself, rather than its distributional effects, that is unfair. I conclude with some considerations in favour of this second view, while accepting that both are valid responses to the problem I describe.

People who care about both personal responsibility and social equality often uphold the Luck Egalitarian principle that “It is bad—unjust and unfair—for some to be worse off than others through no fault [or choice] of their own.” (Temkin 1993: 13). This principle is egalitarian in that it implies, as Arneson puts it, that “everyone should have the same, in some respect, or alternatively that we should bring it about that people reach a condition that is closer to, rather than further from, everyone’s having the same, in some respect” (Arneson 2006: 2). However, it also respects individual responsibility by holding that the respect in which everyone should have the same is that they should be equally well off only in so far as this is a result of their ‘brute luck’, i.e. is not something that they chose or deserved.

One case of brute luck, which should in many ways be a paradigmatic example of what the Luck Egalitarian is seeking to eliminate, is that how well individuals fare often depends upon how well-off their parents are, especially during the period of their birth and upbringing. Since nobody gets to choose their parents, it is nobody’s fault or choice that they are worse off than others for this reason. Yet, parental income has consistently been shown to be one of the principal drivers of how well people fare in adulthood.

To be sure, the problem of such inherited inequality is complex and socially entrenched. Putting aside any concerns that it might reflect some sort of inherited genetic fitness (arguably an undeserved inequality in itself, even if one allowed that such inheritance was possible), the relationship between how well parents and their children fair depends upon a variety of social, cultural and economic factors. However, let us imagine that we already live in a highly egalitarian society in which most or all of these, such as access to education and healthcare, stickiness in the labour market and social and economic prejudice, have already been addressed. Furthermore, since this is a highly egalitarian society, let us imagine that parental inequalities are solely due to differences in what parents chose or deserved. It is still likely that children born to well-off parents will fare better than those born to poorer parents. They will not only have better childhoods, but will go on to be better off for the rest of their lives.

In particular, consider the following case. Two couples start out in a situation of fair equality. One couple, the Prudents, are thrifty and industrious and come to have more than the other couple, the Imprudents, who are fickle and lazy. Let us assume that the ways in which the Prudents were prudent, and the Imprudents were not, make the inequality between them wholly justified on luck egalitarian grounds. What should we say if both couples have a child? Call the Prudents child Little Pru and the Imprudents child Imp Jr. Because the Imprudents are less well off than the Prudents, Imp Jr. will be worse off than Little Pru. This will be the case even if Little Pru and Imp Jr. are equally prudent themselves, and do not act in any other way that might justify the inequality between them. How could this inequality between Imp Jr. and Little Pru possibly be justified on luck egalitarian grounds?

In section 1, I set out why I think that luck egalitarians may be lead to the conclusion that this inequality, though bad in one respect, should nevertheless not be neutralized, because doing so would create an equally unjustified equality between the Prudents and the Imprudents. In section 2, I argue that this conclusion is problematic in at least two respects and that an alternative view about luck egalitarianism would be preferable. Finally, in sections 3 and 4 I offer two such alternatives. Firstly, that luck egalitarians should take a different view of undeserved equalities and undeserved inequalities and secondly that they should switch from objecting to the distributional effects of unequal brute luck to objecting to differential brute luck itself.

## Why Luck Egalitarians May Find Inherited Inequalities Acceptable

According to standard views about luck egalitarianism, differential brute luck is unfair because it moves the distribution of goods away from what individuals would have chosen or deserved. On this view, there is nothing unfair about unequal brute luck per say, but only its distributional effects. This is the view expressed by most canonical statements of luck egalitarianism, such as “a fair distribution of risks and benefits is one that is sensitive to different people’s choices, but insensitive to their brute bad luck” (Dworkin 2000: 451) or “the primary egalitarian impulse is to extinguish the influence on distribution of … brute luck” (Cohen 1989: 908). Accordingly, luck egalitarians are under no imperative to neutralize differential brute luck itself. Instead, their duty is, first and foremost, to produce a final distribution of whatever we take to be valuable that is as insensitive as possible to the effects of unequal brute luck.

However, it is going to be difficult to produce a distribution of resources that is insensitive to the unequal brute luck suffered by Little Pru and Imp Jr. in this case. One obvious approach would be to redistribute resources from the Prudents to the Imprudents for the duration of Imp Jr’s childhood and upbringing. Since the inequality between their parents is the sole cause of the inequality between Imp Jr. and Little Pru, this redistribution is the only way to give the two children an equal start in life.^1^ However, this would not be the only effect of this policy, since it would also make the Imprudents better off than they chose or deserved to be for the duration of Imp Jr’s childhood, in a sense undeservedly compensating them for their imprudence. Similarly, this redistribution would make the Prudents worse off than they would have been, both in absolute terms and relative to the Imprudents, through no fault or choice of their own.

Since redistributing between the two couples makes some worse off, through no fault or choice of their own, someone who viewed the distributional effects of unequal brute luck as the source of its unfairness could object to this, since it makes the distribution of resources no less sensitive to people’s brute luck but only moves these effects around. Rather than Imp Jr. being worse off, through no fault or choice of their own, both the Prudent’s and their child, Little Pru, will be worse off instead. Even if we thought that making Little Pru worse off did not matter, because they did not deserve the good luck of being born to the Prudents, the Luck Egalitarian would still object that Imp Jr’s bad brute luck is unfairly interfering with the ‘justifiably unequal’ distribution of goods between the Prudents and the Imprudents.

The alternative course of action would be to redistribute goods directly to Imp Jr., as compensation for their poor childhood, and to try and minimize any cost this would place on the Prudents, either in absolute terms or relative to the Imprudents. Without physically separating Imp Jr. from their parents, which would be objectionable on other grounds, the best way of achieving this would likely to be a lifelong programme of redistribution from Little Pru to Imp Jr. once they are adults.

## Why Redistributing between Children Is Unsatisfactory

I find this implication of the Luck Egalitarian’s view problematic in this case in two respects. Firstly, it can leave the luck egalitarian with no justifiable response to the clearly unjustified unfairness of inherited inequalities, and secondly, it appears to undermine individual’s responsibility by denying them the opportunity to lead free and equal lives according to their moral aims, even where these are otherwise consistent with luck egalitarianism.

The first of these problems emerges if the redistribution between Little Pru and Imp Jr. was not fully efficient at neutralising the effects of their unequal starts in life. This is not unrealistic since the harms produced by childhood poverty can be long-lasting and hard to overcome. Furthermore, the redistribution may itself be costly to administer and enforce. The generation containing Little Pru and Imp Jr. might then be left worse off if we redistributed between them than they would have been if we had redistributed between their parents instead. In this case, both children can be said to bear a cost, because of Imp Jr’s childhood poverty, which their parents did not have to bare, since they had equal starts in life. Hence, they are made worse off than their parents through no fault or choice of their own.

In this case, a luck egalitarian should seek to compensate the children via further redistribution, if they can. As it was the Imprudents’ imprudence that led to Imp Jr’s childhood poverty, the most obvious policy would be to redistribute between the Imprudents and both Little Pru and Imp Jr. However, it could be that such redistribution would not help, because there is no way of redistributing from the Imprudents, who may be old and have little, without further harming their offspring and so requiring even more, inefficient, redistribution from Little Pru to Imp Jr. to make up for this. Furthermore, it is plausible that the Prudents should also redistribute some of their resources because, while some of their good fortune is due to their prudence, the fact that they do not need to redistribute to anyone in their own generation who has suffered from childhood poverty is not. Therefore, they are better off than their children through no ‘merit or effort of their own’, but simply because of the greater equality of the generation into which they were born. Hence, the Luck Egalitarian still seems to have some reason to redistribute from P, even if their sole goal is to equalise the distributional effects of bad brute luck between Little Pru and Imp Jr.

Finally, redistributing from the Prudents to the Imprudents, rather than to Little Pru and Imp Jr., is likely to be a more efficient way to remove the inequality between these children. If this were the most efficient means of removing the costs facing Little Pru and Imp Jr., then the fact that it also benefits the Imprudents seems like a price worth paying.

From this, we can see that it may be impossible for the Luck Egalitarian who accepts this standard view to produce a fairer outcome via redistribution alone. If we redistribute between Little Pru and Imp Jr., and this redistribution is inefficient, then we create a situation in which these children’s generation is worse off than their parents’ because they had the brute bad luck to grow up under conditions of inequality. However, if we redistribute from the Prudents to the Imprudents, to create equality among their children, then we prevent the Imprudents from facing the full consequences of their imprudence. In both cases, some are made worse off, and there is no way to make the distribution of goods insensitive to Imp Jr’s bad brute luck. This is so despite this brute luck being clearly unjustified and directly neutralizable by redistribution from the Prudents to the Imprudents.

The second problem with the standard view of luck egalitarianism emerges if we consider the moral aims of people in this case, i.e. what sort of world they would like to inhabit. If we only redistributed between Little Pru and Imp Jr., and not between their parents, we could be undermining these aims, even if they were luck egalitarian in spirit.

For instance, it is perfectly reasonable to expect that Little Pru and Imp Jr. would prefer a genuinely equal start in life to an unequal childhood followed by a life of redistribution. Denying them this fair playing field because their parents had to feel the consequences of their own choices demeans both their moral concern for each other and for at least one of theirs parents. One obvious reason for this is that both Little Pru and Imp Jr. may wish that their allocation of resources is sensitive to the choices that they make, but not to any further brute luck in their lives. However, this requires clearly distinguishing between their choices and actions for which they can be held responsible and those for which they cannot. Such a distinction will be far easier to draw, and to implement, if people start out life in more similar positions than if, from the very beginning of their lives, they already face very different burdens of brute luck.

Furthermore, whilst we may reasonably dismiss the fact that the Imprudents would probably prefer to receive some redistribution from the Prudents, and so be able to raise their children well rather than in poverty, we should still take into account the fact that they may reasonably believe it is wrong for their children, or anyone, to be dependent upon others to compensate them for having a poor start in life. Similarly, it is plausible that the Prudents, though satisfied with their current position and what it allows them to do for their children, may reasonably prefer to take on a redistributive burden themselves so that their children can go on to live in a free and equal society, rather than saddling their children with the responsibility of looking after those less fortunate, in return for continuing to reap the rewards of their parents’ prudence.^2^

Surely, luck egalitarians should support moral aims such as these, were they to arise, and not advocate policies that might undermine or contradict them.

In stating this objection, I acknowledge that it does not imply any internal inconsistency in standard luck egalitarianism per say. However, it does conflict with the motivations behind it. Perhaps the luck egalitarian would be all in favour of some voluntary arrangement in situations such as these, under which the Prudents and the Imprudents redistributed between themselves. However, what if such an arrangement was not possible? It might be shameful to either party to give, or receive, charity of this sort or both may fear voluntarily opening themselves up to the moral claims of others if there was a chance that this would not be reciprocated.

Furthermore, even if luck egalitarianism did not undermine the moral aims of the people it affected, it seems demeaning to deny somebody the equal opportunity in life that the luck egalitarian claims to support. By compensating Imp Jr. for their poor childhood, the luck egalitarian may, possibly, achieve their aim of reducing the distributional effects of brute luck. However, if the cost of this is that people must face this bad luck when they did not have to, and only be compensated for it afterwards, this appears to make use of people as a means of producing the fairest distribution of resources, rather than distributing resources so as to respect people’s responsibility to choose what sort of life they want to live.

We should, therefore, consider what alternatives might save the Luck Egalitarian from these problems. However, before doing so, I should note that there may, of course, be many instrumental reasons for not redistributing between the Prudents and the Imprudents. These include the effects of such redistribution on the incentives faced by people to be prudent in future, the desire to punish the Imprudents for their imprudence or their reckless procreation, and the sense that the Prudents simply have no specific duty to help the Imprudents. At the very least, such redistribution is still likely to be somewhat inefficient, since the Imprudents may not make such good use of their resources as the Prudents, even if they were required to use them for the benefit of their children. However, none of these are properly the concerns of luck egalitarianism per say, as I understand it, and hence are not something I will consider. My concern here is solely with the unfairness of unequal brute luck.

## Why Asymmetrical Views about the Badness of Inequality Can Avoid this Result

The problem under discussion emerges from the claim that it is unfair for the Prudents to become worse off, and the Imprudents better off, just because of Imp Jr’s bad brute luck in being born to poor parents. If we wish to preserve this ‘justified’ inequality between the Prudents and the Imprudents, then it seems, I have argued, that we may have no means of compensating Imp Jr. for their bad brute luck in this case that is fully consistent with both the spirit and effectiveness of luck egalitarianism.

However, most statements of luck egalitarianism only make explicit claims about the unfairness of undeserved inequality and leave open the question of whether undeserved equality is equally unfair.

The view that it is not has been defended by Shlomi Segall, who proposes the following ‘asymmetrical’ version of luck egalitarianism: “It is bad for one to be worse off than another through no fault or choice of one’s own. It is never bad, with respect to equality, for one to be equal to another through no merit or effort of her own.” (Segall 2015: 359).

Segal has developed many arguments defending this view, mostly on the basis that it is at least no less reasonable than the standard ‘symmetrical’ view discussed in the previous section (Segall 2012, 2015, 2016: 48–78).

Let me briefly sketch just one of these. As mentioned in the introduction luck egalitarianism supposedly incorporates our concerns for both personal responsibility and social equality. However, Segall points out that in its standard, symmetrical, form Luck Egalitarianism is not actually concerned with inequality at all, but only with how well-off people fair relative to what they chose or deserved. As an example, he points out that any truly egalitarian concern for the worst-off person in an outcome should depend, at least to some extent, on the fact that they actually are the worst-off person in that outcome. However, the standard luck egalitarian view does not do this. Rather it is only concerned about this person’s being worse off than everyone else only because this state is either unchosen or undeserved. This view would have an identical concern for this person even if they were as well of as everyone else, so long as the difference between how well off they are and what they would have chosen or deserved would be the same. Segall’s asymmetrical view, however, is properly sensitive to inequality, in that it would not condemn unchosen or undeserved equalities but remains highly sensitive to choice and desert when some worse are off than others (Segall 2015: 361).

I believe that the case I have sketched in the previous section provides another argument in favour of this Asymmetrical View. Such a view would imply that, whilst the inequality between Little Pru and Imp Jr. is unfair, and therefore bad, the inequality between the Prudents and the Imprudence is no more fair than their equality, and hence that their being made equally well off via redistribution would be no worse, at least with respect to inequality. In other words, on this view both the inequality between the Prudents and the Imprudents and their being made equally well off would be fair, the first because it is a justified inequality and the second because it is an equality.

This allows us to conclude that such a redistribution is acceptable, allowing the luck egalitarian to take direct action to neutralise the bad brute luck that Imp Jr. would otherwise face, which the standard conception of luck egalitarianism would otherwise seem to disallow. The Prudents’s prudence and the Imprudents’s imprudence create a justification for their inequality, but they no longer negate the justification for their equality. The undeserved inequality between Little Pru and Imp Jr., therefore, creates a sufficient justification for redistributing between the Prudents and the Imprudents, which would move towards an outcome that was unambiguously better with respect to equality.

This seems to me like the right conclusion. If luck egalitarians are willing to adopt such an asymmetrical view about the unfairness of inequality, their position might be much more suitable as a principle of intergenerational fairness.

## An Alternative View about the Badness of Unequal Brute Luck

However, there is at least one other possible view about the unfairness of unequal brute luck that would allow us to draw the same conclusion. On this view, just as bad brute luck is intrinsically bad, differential brute luck is intrinsically unfair, and it is this rather than the distributional effects of this brute luck that should be a Luck Egalitarian’s primary concern. Since it is unfair that bad things happen to some and not to others, luck egalitarians should, therefore, seek, first and foremost, to neutralise bad brute luck, and only then, if this proves impossible or ineffective, to equalise its distributional effects by way of compensation.

I believe that this conception of the Luck Egalitarian’s primary concern would also allow us to escape the problems set out in section 2. This is because, if unequal brute luck is intrinsically unfair, then we should neutralise it, even in cases where this means redistributing goods between agents who are not themselves the subjects of unequal brute luck. This can be easy to miss since it is only relevant to cases in which the distribution of resources is itself a source of brute luck. However, since it involves inherited inequality, this is just such a case, and hence allows us to differentiate between this view and its more standard counterpart. In our case, since Imp Jr’s bad brute luck is merely a function of their parents poverty we should redistribute goods from the Prudents to the Imprudents for the duration of Imp Jr’s childhood, so that they no longer suffer the bad brute luck of their parents poverty, but can have the same start in life as Little Pru.

This view has not, I think, been much discussed in the literature thus far. Part of the reason for this may be that that it gets mistaken for another view, that luck egalitarians should seek to neutralize brute luck in general. As Elizabeth Hurley has demonstrated, this other view is false; luck egalitarians should not object to everybody enjoying good brute luck and would not object, any more than utilitarians or many other moral theories, to everybody suffering the same bad brute luck (Hurley 2003: 156). However, the view I am discussing has no such implications. Since these cases involve no differential brute luck, and hence, whilst they may be good or bad, there is nothing unfair about them on this view.^3^

Another reason for the lack of discussion about this view could be that in most cases the difference between this and the standard luck egalitarian view is insignificant and uncontroversial. For instance, in the case of a preventable disaster where we have to decide between acting to prevent the disaster at some cost or compensating people after it has taken place, both views will lead us to conclude that we should do whatever will most efficiently and effectively prevent people being harmed by the disaster, relative to those who were unaffected. Indeed, this view is more similar to the standard Luck Egalitarian view than Segall’s Asymmetrical View, since it would still condemn equalities that resulted from differential brute luck as no less unfair than inequalities that emerged from the same brute luck. Yet the view does offer an alternative response to Segall’s charge that symmetrical luck egalitarianism is not truly egalitarian, since it implies that the luck egalitarians concern should not be about the distribution of resources at all but is, quite literally, a concern to equalize the distribution of, brute, luck.

The other difference between this view and Segall’s Asymmetrical View is that while Segall’s view implies that equality between people is always justified, my view would still hold that equalities can be unjustified, where they result from unequal distributions of brute luck. It is simply that, in the case under discussion, an egalitarian concern to neutralize the bad brute luck faced by Imp Jr. should be the Luck Egalitarians overriding concern, regardless of its wider distributional effects. This justifies the, otherwise unjustifiable, equalization of resources between the Prudents and the Imprudents. Hence, while both views see redistribution from the Prudents to the Imprudents as justified on the grounds of fairness in this case, Segall’s view would also imply that it would be acceptable even if these couples had never had children. This more extreme view is one I find harder to accept, as it appears overly insensitive to differential effort and desert. For this reason, I prefer this second view, that differential brute luck is intrinsically unfair, but its distributional effects are not.

## Conclusion

What is unfair about unequal brute luck? In this paper, I have considered three possibilities: that its effects on the distribution of resources are always unfair, that its effects on the distribution of resources are unfair only when they produce inequalities and that unequal brute luck is, itself, intrinsically unfair. I have argued that, in certain intergenerational cases in which the choices of one generation lead to the differential brute luck of the next generation, the first of these views has problematic implications, unacceptably limiting what kinds of response a Luck Egalitarian might take to neutralizing this injustice for the second generation and potentially undermining people’s moral aims. I suggest that the second two accounts of why unequal brute luck is unfair can both escape these problems, but that they will have different implications for other kinds of case. Ultimately, I find the view that unequal brute luck is intrinsically unfair to be the more acceptable of these accounts, although my arguments to that end are far from exhaustive. I think these cases pose fascinating challenges for egalitarians that I hope may receive greater attention in the future.

## Footnotes

1 Given the highly egalitarian nature of the society under consideration, we must assume that other sources of inequality, such as unequal access to education and healthcare, have already been removed. Therefore, unlike in most real-world cases, further interventions in the provision of these services will not rectify the inequality between these children.

2 Note that the option taken by many wealthy parents, of simply shirking their distributional responsibilities and encouraging their children to do the same, is simply not an option in the egalitarian paradise assumed in this case.

3 It may be objected that the view would advocate imposing additional bad brute luck on people to create a more equal distribution of luck on a whole. However, note that the unfairness of differential brute luck is predicated on the badness of bad brute luck. This implication is, therefore, the exact analogue of the famous ‘Levelling Down Objection’ which faces many accounts of inequality and to which this view is no more vulnerable than others
